# Draft Genome Sequence of *Lactobacillus rhamnosus* CRL1505, an Immunobiotic Strain Used in Social Food Programs in Argentina

**DOI:** 10.1128/genomeA.00627-13

**Published:** 2013-08-15

**Authors:** María Pía Taranto, Julio Villena, Susana Salva, Susana Alvarez, Graciela Savoy de Giori, Graciela Font de Valdez, Elvira M. Hebert

**Affiliations:** Centro de Referencia para Lactobacilos (CERELA-CONICET), San Miguel de Tucumán, Argentina

## Abstract

We report the draft genome sequence of the probiotic *Lactobacillus rhamnosus* strain CRL1505. This new probiotic strain has been included into official Nutritional Programs in Argentina. The draft genome sequence is composed of 3,417,633 bp with 3,327 coding sequences.

## GENOME ANNOUNCEMENT

*Lactobacillus rhamnosus* is used in the manufacture of cheese and other dairy products to aid ripening and enhance flavors. This organism has also been shown to stimulate the immune system and have antibacterial activity against intestinal pathogens, indicating that it may be useful as a probiotic, which is defined as a live microorganism that, administered in adequate amounts, confers a health benefit to the host (1). Clancy (2) suggested the term “‘immunobiotic’” to identify a bacterium that promotes health targeting on the mucosal immune system. Most probiotic organisms are lactic acid bacteria (LAB), which induce a long list of health benefits (3).

In a previous work, Salva et al. (4) demonstrated that *Lactobacillus rhamnosus* strain CRL1505 isolated from goats’ milk stimulates the innate and adaptive immune response of the common mucosal system in a dose-dependent way and confers resistance to infection with *Salmonella enterica* serovar Typhimurium and *Streptococcus pneumoniae* in immunocompetent mice. Moreover, this immunobiotic strain confers resistance to infection with respiratory syncytial virus in adult and infant mice (5). In human trials, Villena et al. (6) showed that the consumption of a fermented dairy product containing *L. rhamnosus* CRL1505 is associated with a significant decrease in the duration and severity of mucosal infections, providing the first evidence that a fermented dairy product containing this immunobiotic strain may have a beneficial effect against respiratory infections in young children. Based on the results summarized above and given the high morbidity and mortality associated with infectious diseases of the airway in children, dietary intervention using a dairy product containing the probiotic CRL1505 strain can be useful to improve the health status of this vulnerable population. On the basis of these results, this new probiotic strain has been included into official National Nutritional Programs in Argentina. Since 2008, the probiotic yogurt containing *L. rhamnosus* CRL1505 (Yogurito) is given daily to more than 200 thousand children in Tucumán, Argentina.

The genomic DNA was extracted from the cultured bacterium according to the method reported by Pospiech and Newman (7). Whole-genome sequencing of *L. rhamnosus* CRL1505 was performed with a 454 GS Titanium pyrosequencer at INDEAR, Argentina. Genomic libraries containing 8-kb inserts were prepared, and 257,911 paired-end reads and 400,821 single-end reads were generated using the 454 GS system, giving 42-fold coverage of the genome. Approximately 98.7% of these reads were assembled into 47 large scaffolds, including 173 nonredundant contigs, using version 2.6 of the 454 Newbler assembler (454 Life Sciences, Branford, CT). The draft genome is a single circular chromosome of 3,417,633 bases in length, with a mean GC content of 46.3%. Genome annotation was performed by the standard operating procedures (SOPs) for prokaryotic annotation from ISGA (8), the RAST annotation server (9), the Glimmer 3.02 modeling software package (10), tRNAscan-SE 1.21 (11), and RNAmmer 1.2 (12). A total of 3,327 coding sequences (CDS), 40 structural tRNAs, and 5 rRNA operons were predicted. There are 314 RAST subsystems represented in the chromosome.

### Nucleotide sequence accession numbers.

This whole-genome shotgun project has been deposited at DDBJ/EMBL/GenBank under the accession ATBI00000000. The version described in this paper is version ATBI00000000.1.
